# MOBILE-BASED COGNITIVE TRAINING IN CHINESE AMERICANS: PERSPECTIVES FROM OLDER ADULTS AND ADULT CHILDREN

**DOI:** 10.1093/geroni/igad104.3708

**Published:** 2023-12-21

**Authors:** Tingzhong (Michelle) Xue, Eleanor McConnell, Bei Wu, Hanzhang Xu

**Affiliations:** Duke University School of Nursing, Durham, North Carolina, United States; Duke University School of Nursing, Durham, North Carolina, United States; New York University, New York City, New York, United States; Duke University, Durham, North Carolina, United States

## Abstract

The population of older Chinese Americans is growing rapidly. However, their access to culturally responsive dementia prevention services is very limited. Cognitive training may delay cognitive decline in older adults, but such training has not been widely accessible to older Chinese Americans. This study explored perspectives of older Chinese Americans and their adult children on dementia prevention and cognitive stimulating activities to inform co-design of a culturally appropriate, mobile-based cognitive training for this population. We conducted five focus groups in Mandarin using Zoom video conferencing, with four groups of older Chinese Americans (n=21) and one group of adult children (n=6). We applied rapid qualitative analysis, guided by the Health Belief Model. We identified seven themes. Overall, participants had modest knowledge of dementia, and viewed health care professionals as credible information sources for dementia prevention. Some participants thought any social activity would provide cognitive stimulation. Both older adults and adult children expressed strong interest in cognitive training but were concerned about losing interest over time and transportation and language barriers. Both groups noticed similar advantages (e.g. benefits for cognition, interesting exercises), and disadvantages (e.g. eyestrain, addiction) of mobile-based cognitive training but had different perspectives on acquiring health information online. Adult children were eager to provide support for older adults to participate in cognitive training, whereas older adults preferred to engage in cognitive training autonomously. Knowledge generated from this study forms the foundation for designing of a tailored mobile-based intervention to prevent cognitive decline among older Chinese Americans.

